# Diagnosis of foot-and mouth disease by real time reverse transcription polymerase chain reaction under field conditions in Brazil

**DOI:** 10.1186/1746-6148-4-53

**Published:** 2008-12-31

**Authors:** Tatiane A Paixão, Alcina V Carvalho Neta, Naimes O Paiva, Jorge R Reis, Meirivan S Barbosa, Claudia V Serra, René R Silva, Tammy R Beckham, Barbara M Martin, Neville P Clarke, L Garry Adams, Renato L Santos

**Affiliations:** 1 Departamento de Clínica e Cirurgia Veterinária, Escola de Veterinária da Universidade Federal de Minas Gerais, Belo Horizonte, MG, Brazil; 2 Laboratório Nacional Agropecuário – Pará, Belém, PA, Brazil; 3 Laboratório Nacional Agropecuário – Minas Gerais, Pedro Leopoldo, MG, Brazil; 4 Texas Veterinary Medical Diagnostic Laboratory, College Station, TX, USA; 5 National Veterinary Services Laboratories, U.S. Department of Agriculture, Animal Plant Health Inspection Service, Center for Veterinary Biologics, Ames, IA, USA; 6 National Center of Excellence for Foreign Animal & Zoonotic Diesease Defense, College Station, TX, USA; 7 Department of Veterinary Pathobiology, College of Veterinary Medicine, Texas A&M University, College Station, TX, USA

## Abstract

**Background:**

Foot-and-mouth disease (FMD) is an economically important and highly contagious viral disease that affects cloven-hoofed domestic and wild animals. Virus isolation and enzyme-linked immunosorbent assay (ELISA) are the gold standard tests for diagnosis of FMD. As these methods are time consuming, assays based on viral nucleic acid amplification have been developed.

**Results:**

A previously described real-time reverse transcriptase polymerase chain reaction (RT-PCR) assay with high sensitivity and specificity under laboratorial and experimental conditions was used in the current study. To verify the applicability of this assay under field conditions in Brazil, 460 oral swabs from cattle were collected in areas free of FMD (n = 200) and from areas with outbreaks of FMD (n = 260). Three samples from areas with outbreaks of FMD were positive by real-time RT-PCR, and 2 of those samples were positive by virus isolation and ELISA. Four other samples were considered inconclusive by real-time RT-PCR (threshold cycle [Ct] > 40); whereas all 200 samples from an area free of FMD were real-time RT-PCR negative.

**Conclusion:**

real-time RT-PCR is a powerful technique for reliable detection of FMDV in a fraction of the time required for virus isolation and ELISA. However, it is noteworthy that lack of infrastructure in certain areas with high risk of FMD may be a limiting factor for using real-time RT-PCR as a routine diagnostic tool.

## Background

Foot-and-mouth disease (FMD) is a highly contagious viral disease that affects cloven-hoofed domestic and wild animals. Foot-and-mouth disease is considered the most economically important animal disease worldwide, and outbreaks of FMD require immediate notification to the World Organization for Animal Health (OIE). Outbreaks of FMD result in sanitary barriers that prevent export of bovine and swine products [1]. Furthermore, FMD causes enormous losses to the animal industry due to costs associated with control or eradication measures, including massive vaccination and/or destruction of infected herds, as well as decreases in milk and beef production as a result of clinical disease. Clinically affected cattle have vesicular lesions in the oral cavity, foot, and udder, which are associated with fever, lameness, salivation, and anorexia. Foot-and-mouth disease usually has a high morbidity and low mortality, with mortality occurring mostly in young animals [2].

In Brazil, the number of cases of FMD has been steadily decreasing since the 1980s [3]. The national program for control and eradication of FMD (Programa Nacional de Controle e Erradicação de Febre Aftosa [PNEFA]), implemented in 2001, has resulted in recognition by the OIE of vast areas of the Brazilian territory as free of FMD with vaccination [4]. However, the recognition of most of these areas was suspended due to the outbreak of FMD in the State of Mato Grosso do Sul in 2005, from which part of the biological samples used in the current study was obtained.

The *Foot-and-mouth disease virus *(FMDV; family *Picornaviridae*, genus *Aphthovirus*) has a single-stranded, positive-sense RNA genome of approximately 8.4 Kb. There are 7 serotypes with a large number of variants spread over several regions in the world [1,2]. Serotypes A, O, and C have been detected in Brazil [3]. Considering that FMD is highly contagious and has clinical signs similar to other vesicular diseases, a quick definitive diagnosis is extremely important [5].

A presumptive clinical diagnosis associated with laboratory tests such as serology, virus isolation, and antigen detection are the basis for the diagnosis at the herd level. Serological tests (i.e., virus neutralization and liquid phase enzyme-linked immunosorbent assay [ELISA]) are not time-consuming, but they are indirect tests that do not always allow for differentiation between infected and vaccinated cattle due to the use of poor vaccine or previous infection in endemic areas. Serological techniques are not the technique of first choice to detect an acute infection [6,7]. A definitive diagnosis is based on detection of virus in fluids or epithelium from vesicular lesions or esophageal-pharyngeal fluid collected with a probang [5,7]. Virus isolation is the most reliable diagnostic method, but it is labor-intensive, time-consuming, and requires properly equipped facilities. Sandwich ELISA is a much faster approach to detect viral antigens, but it has low sensitivity, so its primary indication is to confirm and type the FMDV after isolation in cell culture [5]. Therefore, several groups have been developing faster diagnostic methods for FMD based on amplification of specific sequences of the viral genome by reverse transcription polymerase chain reaction (RT-PCR) [8-21], which can be applied to different kinds of biological samples such as fluids and tissues, and in some cases, this approach allows identification of infected animals even before development of clinical signs or positive virus isolation as well as identification of positive cattle at the end of the course of infection when virus isolation may be negative [12,22]. In addition, in a study in which results were compared between reference laboratories, RT-PCR results were more consistent among laboratories as compared to virus isolation and ELISA results, which varied from low to high sensitivity when aliquots of the same samples were processed by 5 different laboratories [16]. Reverse transcription polymerase chain reaction is also useful for typing FMDV isolates [23]. However, conventional RT-PCR does not have optimal results in terms of specificity and sensitivity [1]. Thus, recently, a method based on real-time RT-PCR amplification and a fluorescent probe resulted in significantly improved sensitivity and specificity under experimental conditions [12]. A previously described real-time RT-PCR method was employed in the current study, which demonstrated high specificity and sensitivity under laboratory and experimental conditions [12], for the diagnosis of FMD under field conditions in Brazil.

## Methods

### Source of samples

A total of 460 oral swabs from adult cattle of both sexes were used in this study. These samples originated from different regions according to the following groups. Group 1 consisted of 200 healthy cattle from an area free of FMD with vaccination (101 samples from Pedro Leopoldo, State of Minas Gerais; 99 samples from Igarapé, State of Minas Gerais). Group 2 consisted of 60 cattle from Carero da Várzea, State of Amazonas (region classified as "unknown risk for FMD"), which were sampled during an outbreak of FMD (type C virus) in 2004 [24]. These cattle did not have any clinical signs and were sampled more than 30 days after the identification of the index case. Group 3 consisted of 200 cattle suspected of FMD (some with clinical changes compatible with FMD from Eldorado and Japorã, State of Mato Grosso do Sul). These swabs were obtained during an outbreak of FMD (type O virus) in 2005 [4].

### Sampling

Dacron-tipped swabs were obtained from the oral cavity by swabbing the oral mucosa and tongue, saturating the swab with saliva while avoiding contamination with ingested material. Immediately after sampling, the swabs were placed into a 2-ml sterile cryogenic tube containing 1.5 ml of Dulbecco's Minimal Essential Medium [DMEM] (Gibco^®^, Invitrogen Corp., Carlsbad, CA) with antibiotic and antimycotic (Gibco^®^). The tubes containing the swabs from cattle from an area free of FMD (State of MinasGerais) were immediately frozen on dry ice and stored at -70°C until further processing. However, due to the unavailability of dry ice in the areas of the outbreaks (Eldorado and Japorã in the State of Mato Grosso do Sul, and Carero da Várzea in the State of Amazonas) some samples were kept at 4°C for up to 7 days prior to freezing and storage at -70°C.

### RNA extraction

RNA samples were extracted using the Mini RNeasy kit (Qiagen Inc., Valencia, CA) following the manufacturer's instructions. Briefly, the tubes containing the swabs were thawed and thoroughly homogenized by vortexing, and 140 μl of the medium were added to 540 μl of the RLT buffer that is included in the kit, followed by 700 μl of 70% ethanol, and then transferred to a Mini RNeasy column previously inserted into a 2-ml collecting tube. RNA was immobilized in the column by centrifugation, sequentially washed, and eluted in 40 μl of RNase-free water.

In addition to the RNA samples obtained from oral swabs as described above, RNA samples were purified using Trizol (Invitrogen Corp., Carlsbad, CA.) from 3 reference strains (O1 Campos, A24 Cruzeiro, and C3 Indaial) representing the 3 serotypes that occur in Brazil (A, O, and C), and 2 previously characterized field isolates (FMDV serotypes A and O, isolated from outbreaks in the states of Roraima in 1999 and Mato Grosso do Sul in 2005). These RNA samples were used as positive controls.

### Real-time reverse transcription polymerase chain reaction

The rRT-PCR amplification protocol used in the current study was previously described [12]. This method amplifies a conserved segment of the FMDV RNA polymerase gene (3D; GenBank AF189157). The following primers were used 5'-ACTGGGTTTTACAAACCTGTGA-3' and 5'-GCGAGTCCTGCCACGGA-3' along with the probe 5'-TCCTTTGCACGCCGTGGGAC-3' labeled with 6-carboxyfluorescein at the 5' end, and the quencher tetramethyl rhodamine at the 3' end. This method amplifies the RNA from all serotypes of FMDV, but does not amplify nucleic acids from other viruses that cause vesicular diseases [12]. Reactions were performed using 25 μl Cepheid tubes (Cepheid Inc., Sunnyvale, CA) containing all the dehydrated reagents required for amplification of the FMDV RNA (Vet-Alert, Foot-and-Mouth Disease, Tetracore Inc., Gaithersburg, MD) that were rehydrated with 22.5 μl of rehydration buffer (Vet-Alert). As controls, 2.5 μl of RNA samples or an oligonucleotide used as positive control (Vet-Alert) or TE buffer (Tris-ethylenediamine tetra-acetic acid [EDTA]) instead of RNA as a negative control, were added to the respective reaction tubes. The real-time RT-PCR reactions were carried out in a Smart Cycler II thermocycler (Cepheid Inc.). The one-step real-time RT-PCR amplification started with reverse transcription for 1 hr at 60°C, followed by PCR with the following parameters: 55 cycles of 2 sec at 95°C and 30 sec at 60°C. One positive and 1 negative control were included in each reaction.

### Virus isolation

The samples (medium containing the swabs) were filtered using a 0.2-μm filter, and 500 μl was then inoculated onto a monolayer of IBRS (Instituto Biologico Rim Suino)-2 cells grown in 25-cm^2 ^flasks and kept without washing for the duration of the assay (up to 72 hr). After inoculation, the cultures were checked for cytopathic effects (CPE) every 24 hr for 72 hr. Cultures that did not develop CPE were lysed by freezing at -70°C and reinoculated (500 μl) onto a new monolayer of IBRS-2 cells, which was then evaluated for an additional 72 hr. Cultures with CPE were stored at -70°C until processing for indirect sandwich ELISA.

### Indirect sandwich enzyme-linked immunosorbent assay

Samples from cultures that had CPE were tested by indirect sandwich ELISA, according to the protocol recommended by the Pan American Foot-and-Mouth Disease Center (PANAFTOSA). Briefly, anti-FMDV serotypes A, O, and C rabbit antiserum was incubated in ELISA plates for 18 hr at 4°C. Antiserum was generated by using a pool of inactivated virus subtypes O1 Campos, A24 Cruzeiro, and C3 Indaial as antigen. The plate was washed and incubated with 1% ovalbumin for 1 hr at room temperature. After washing, the reference antigens and testing samples were added to the wells and incubated for 1 hr at 37°C under agitation. The plate was then washed again and incubated with Guinea pig anti-FMDV (serotypes A, O, and C) antiserum for 30 min at 37°C under agitation, followed by washing and incubation with an anti-Guinea pig antibody conjugated with peroxidase for 30 min at 37°C under agitation. The plate was washed and incubated with a solution of hydrogen peroxide (0.012% H_2_O_2 _in 0.1 M of citric acid, 0.2 M of sodium phosphate, and 0.04% orthophenyldiamine) as a substrate for 15 min at room temperature, and read in an ELISA reader at 492 nm.

## Results

**Figure 1:**
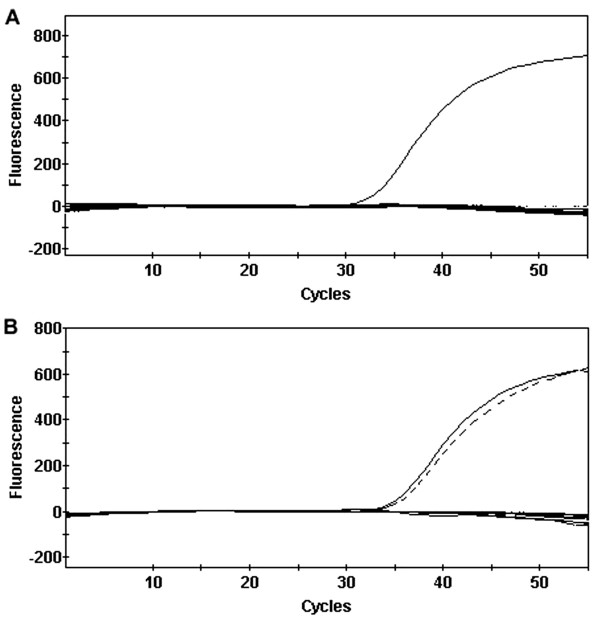
**Representative results of real-time reverse transcription polymerase chain reaction**. A, samples from an area free of foot-and-mouth disease with vaccination, with amplification only of the positive control; B, samples from an area of outbreak (State of Mato Grosso do Sul, 2005), with amplification of 1 positive sample (dashed line) in addition to the positive control (solid line).

Representative results of real-time RT-PCR reactions with samples from the area free of FMD and from areas with outbreaks of FMD are illustrated in Figure 1. All 200 cattle from the area free of FMD with vaccination were negative by real-time RT-PCR and combined virus isolation/ELISA. The 60 samples from Carero da Várzea (State of Amazonas) were negative by real-time RT-PCR and combined virus isolation/ELISA. Among the 200 samples from Eldorado and Japorã (State of Mato Grosso do Sul), 3 were positive by real-time RT-PCR, 2 of which were also positive by combined virus isolation/ELISA; whereas 4 samples were considered inconclusive with cycle threshold (Ct) values higher than 40. Samples considered inconclusive by real-time RT-PCR were all negative by combined virus isolation/ELISA. Importantly, no false-negative results by RT-PCR were observed since none of the samples was positive by virus isolation/ELISA and negative by real-time RT-PCR. The results are summarized in Table 1. The samples from the 3 reference strains (serotypes A, O, and C) and the 2 previously characterized field isolates (serotypes A and O) were all positive by real-time RT-PCR with low Ct values indicating high concentration of viral RNA (Table 2).

**Table 1:** Positive and inconclusive results by real-time polymerase chain reaction and the corresponding virus isolation and enzyme-linked immunosorbent assay (ELISA) results.*

		Virus isolation	
Sample no.	Ct value	CPE	ELISA
527	42.22	Negative	ND
567	44.26	Negative	ND
605	44.26	Positive	Negative
613	34.94	Positive	Negative
652	41.52	Negative	ND
674	36.60	Positive	Positive (type O)
675	35.20	Positive	Positive (type O)

* Origin of samples was Eldorado/Japorã, State of Minas Gerais. Ct (cycle threshold) corresponds to the number of cycles required for a given sample to reach the threshold above which it is considered positive. A sample was considered positive when the Ct value was lower than 40, and inconclusive when the Ct value was higher than 40. CPE = cytopathic effect; ND = not determined. Only samples that resulted in CPE were processed for indirect sandwich ELISA.

**Table 2:** Reverse transcription polymerase chain reaction results from 3 reference strains and 2 previously characterized field isolates.

Sample no.	Description	Serotype	Ct values*
P7	Reference strain – O1 Campos	O	14.53
P8	Reference strain – A24 Cruzeiro	A	13.17
P9	Reference strain – C3 Indaial	C	34.92
P10	Field isolate (Mato Grosso do Sul, 2005)	O	17.12
P11	Field isolate (Roraima, 1999)	A	18.50

* Ct (cycle threshold) corresponds to the number of cycles required for a given sample to reach the threshold above which it is considered positive. A sample was considered positive when the Ct value was lower than 40.

## Discussion

The real-time RT-PCR method used in the current study has proven to be highly sensitive and specific under laboratory and experimental conditions (Callahan et al. 2002), which is supported by our results as all previously characterized field isolates resulted in positive reactions with low Ct values. The 2 samples that were positive by combined virus isolation/ELISA were also positive by real-time RT-PCR (Table 1). In addition, neither false-positives nor false-negatives were detected. Indeed, a recent study demonstrated that this method performed similarly to another real-time RT-PCR protocol for amplification of the 5' untranslated region (5'UTR) of the FMDV genome [22].

The recommended protocol for storage of samples prior to RNA extraction was strictly followed in the case of swabs collected from the area free of FMD with vaccination (State of Minas Gerais). However, it was not possible to supply dry ice to the areas affected by FMD outbreaks, which resulted in storage of samples at 4°C up to 7 days. Presumably, this inadequate procedure for storage of samples likely resulted in a decrease in the sensitivity of the test. However, samples from the area free of FMD, which were properly stored, were all negative by real-time RT-PCR as well as by virus isolation and ELISA, indicating a high specificity of this test as previously demonstrated [12]. A comprehensive recent study of specificity of this method confirmed a very high specificity, but even with the inconclusive reactions considered false-positives, the specificity was 99.9%, which may provide enough accuracy for a diagnosis during an outbreak, but may not be specific enough for surveillance in FMD-free areas [25].

In spite of inadequate storage of samples from cattle suspect of FMD, 3 positive animals were identified by real-time RT-PCR, whereas only 2 of those samples were positive by combined virus isolation/ELISA, indicating comparable sensitivity between these two diagnostic methods, which is in good agreement with previous [12,22]. In contrast, none of the 60 samples collected from cattle without lesions more than 1 month after identification of the index case were positive by real-time RT-PCR. Although these samples were also inadequately stored, negative results are likely to occur in cattle that recovered from clinical lesions since the likelihood of virus isolation is extremely reduced with more than 7–10 days after the appearance of gross lesions [1].

## Conclusion

In summary, the current study supports the notion that real-time RT-PCR is a powerful technique for reliable detection of FMDV in a fraction of the time required for combined virus isolation/ELISA. However, it is noteworthy that lack of infrastructure in certain areas with high risk of FMD may be a limiting factor for using real-time RT-PCR as a routine diagnostic tool.

## Authors' contributions

TAP, AVCN, and RLS collected samples in FMD-free areas; NOP collected samples in areas of outbreaks; JRR performed virus isolation procedures; MSB performed ELISAs; TAP, AVCN, RRS, and RLS did the real time RT-PCR reactions; TRB, BMM, NPC, LGA, and RLS designed the study, analyzed the data, and supervised all procedures; LGA and RLS coordinated the group; and all co-authors were involved in writing and revising the manuscript.
